# Prevalence of human papillomavirus type-18 in head and neck cancer among the Chinese population: A PRISMA-compliant meta-analysis

**DOI:** 10.1097/MD.0000000000014551

**Published:** 2019-02-22

**Authors:** Funa Yang, Yulin Yin, Peng Li, Xiaojun Zhang, Defeng Chen, Yang Liu, Jian Wang, Lanwei Guo

**Affiliations:** a Nursing Department, The Affiliated Cancer Hospital of Zhengzhou University, Henan Cancer Hospital, Zhengzhou; b Department of Head and Neck Surgery, National Cancer Center/National Clinical Research Center for Cancer/Cancer Hospital, Chinese Academy of Medical Sciences and Peking Union Medical College, Beijing; c Henan Office for Cancer Control and Research; d Department of Head Neck and Thyroid Surgery, The Affiliated Cancer Hospital of Zhengzhou University, Henan Cancer Hospital, Zhengzhou; e Office of Cancer Screening, National Cancer Center/National Clinical Research Center for Cancer/Cancer Hospital, Chinese Academy of Medical Sciences and Peking Union Medical College, Beijing, China.

**Keywords:** China, head and neck cancer, human papillomavirus, meta-analysis

## Abstract

**Background:**

China has a high burden of head and neck cancer globally and oncogenic human papillomavirus (HPV) has been hypothesized as a risk factor for head and neck cancer, but research was absent for establishing HPV prevalence in China. We aimed to conduct a meta-analysis to estimate the high-risk HPV-18 prevalence of head and neck cancer in the Chinese population.

**Methods:**

This meta-analysis was reported following the guideline of PRISMA. The reports on HPV and head and neck cancer in a Chinese population published between Jan 1, 2006 and May 31, 2018 were retrieved via CNKI/WANFANG/MEDLINE/EMBASE/COCHRANE databases. A random-effect model was used to calculate pooled prevalence and corresponding 95% confidence intervals.

**Results:**

A total of 1881 head and neck cancer cases from 19 studies were included in this meta-analysis. Overall, the pooled HPV-18 prevalence among head and neck cancer cases was 6.0% (4.1%–7.9%) in China, 31.2% (13.0%–49.4%) in laryngeal cancer, 7.2% (3.9%–10.5%) in oral cancer and 0.6% (0.0%–1.3%) in oropharyngeal cancer, 18.7% (6.2%–31.2%) in fresh or frozen biopsies and 4.3% (2.5%–6.1%) in paraffin-embedded fixed biopsies, 29.5% (15.6%–43.3%) by E6/E7 region and 3.9% (0.5%–7.4%) by L1 region of HPV gene. The highest HPV-18 prevalence was found in Central China.

**Conclusions:**

High prevalence of HPV-18 was found in the samples of Chinese head and neck cancers. Prophylactic HPV-vaccination may reduce the burden of HPV-related head and neck cancer in China.

## 1 Introduction

As a member of the papillomavirus family of viruses, human papillomavirus (HPV) can infect humans by attacking the squamous cell of skin and mucous membranes, including those of the cervix, anogenital region, and head and neck.^[1–3]^ As we all know, nasopharyngeal carcinoma is related to EB virus infection. Except that, about 70% of the remaining squamous cell carcinomas of head and neck are caused by high-risk HPV infection,^[4]^ and the incidence of such subtypes is increasing year by year.^[5]^ However, the frequency of HPV infection in head and neck carcinomas varies between 3% and 84% in different studies.^[6]^ Based on the different nucleotide sequences, HPV can be divided into more than 200 genotypes by DNA sequencing, of which HPV16 and HPV18 as main high-risk types are more closely linked with malignant tumors.^[7,8]^ However, unlike cervical carcinogenesis, the role of HPV-16/18 in the head and neck carcinogenesis has not been clearly defined.

Even in the same country, the HPV prevalence in head and neck cancers range widely.^[9]^ Demographic and racial factors, sample condition, cancer location, and the viral detection method have been proposed to identify possible causes of differences in the results. However, as one of the second common subtype of HR-HPV, the HPV-18 prevalence in China has not been estimated so far.

The aim of the meta-analysis is to estimate the prevalence of HPV-18 detected in head and neck cancer cases and the influence of regions, different cancer sites, specimen types and detection methods in China from all published studies of English and Chinese language literature.

## 2 Materials and methods

The methods were carried out in accordance with the approved guidelines. This study was approved by the ethics committee of Henan Cancer Hospital.

### 2.1 Literature search strategies

The key words “human papillomavirus”, “papillomavirus infections”, “head and neck neoplasms”, “head and neck cancer”, and “head and neck carcinoma” in Chinese language or in the English language were used in combination to search. The retrieved databases included MEDLINE (via PubMed), Excerpta Medica database (EMBASE), Cochrane database, Chinese National Knowledge Infrastructure (CNKI), and Wanfang Data Knowledge Service Platform. Date of the literature was specified between Jan 1, 2006 and May 31, 2018. Additional studies were also identified using cross-referencing.

### 2.2 Inclusion and exclusion criteria

The literature included in the present study meets the following criteria:

(a) to use polymerase chain reaction (PCR)-based methods (including broad-spectrum PCR primers, type-specific PCR primers, or a combination of both kinds of primers) or in situ hybridization (ISH) to amplify HPV DNA,
(b) to report the prevalence of HPV-18 in head and neck cancer tissue samples,
(c) have control group.

The literature excluded in this study were mainly due to the following reasons: were cellular or animal studies; were not conducted in Chinese; necessary data could not be extracted or calculated directly from the original article; reviews; the literature include the repeated data.

### 2.3 Quality assessment

We used a bias risk assessment tool proposed by Hoy D^[10]^ to assess the quality of included studies. The tool including 11 items with a total of 12 scores: External validity

1. (Was the study's target population a close representation of the national population in relation to relevant variables?
2. Was the sampling frame a true or close representation of the target population?
3. Was some form of random selection used to select the sample, or was a census undertaken?
4. Was the likelihood of nonresponse bias minimal? And Internal validity
5. Were data collected directly from the subjects (as opposed to a proxy)?
6. Was an acceptable case definition used in the study?
7. Was the study instrument that measured the parameter of interest shown to have validity and reliability?
8. Was the same mode of data collection used for all subjects?
9. Was the length of the shortest prevalence period for the parameter of interest appropriate?
10. Were the numerator(s) and denominator(s) for the parameter of interest appropriate?
11. Summary item on the overall risk of study bias.)

### 2.4 Data extraction

The data related to the meta-analysis were extracted by 2 independent authors (YFN and PL) based on a standardized data extraction program: first author's name, publication year, geographical areas, cancer site, clinical stage, numbers of cases, and HPV positive cases, HPV test method, specimen types (formalin-fixed paraffin-embedded biopsies [FFPE], fresh or frozen biopsies [FF]).

### 2.5 Statistical analysis

Overall pooled point estimate and 95% confidence interval (95% CI) for HPV-18 prevalence were calculated through the method of DerSimonian and Laird^[11]^ using the assumptions of a random-effects model. For studies with multiple HPV types infection (including HPV-18), the multiple HPV types were separated into different types and the HPV-18 type-specific prevalence represents types for cases with either single HPV-18 infection and multiple HPV-18 infections.

Cochrane *Q* test *(P* <.10 indicated a high level of statistical heterogeneity) and *I*^2^ (values of 25%, 50%, and 75% corresponding to low, moderate and high degrees of heterogeneity, respectively) was used to assess the heterogeneity between eligible studies.^[12]^ Subgroup analyses for HPV-18 prevalence were subsequently carried out according to the geographical areas of the study origin, cancer site, year of publication, number of patients, HPV detection method, types of specimen, and quality score. In the eligible studies, 2 studies contained different cancer sites, 2 studies contained different methods of HPV test and 3 studies contained different types of specimen. For these studies, we treated them as the separate studies and pooled them into appropriated groups when performing stratified analysis. Funnel plot and Begg adjusted rank correlation test for funnel plot asymmetry were performed to test any existing publication bias.

The statistical analyses were performed using STATA SE version 15.0 for Windows (StataCorp LP, College Station, TX). *P* <.05 with 2-tailed was considered statistically significant.

## 3 Results

### 3.1 Systematic review and study characteristics

**Figure 1:**
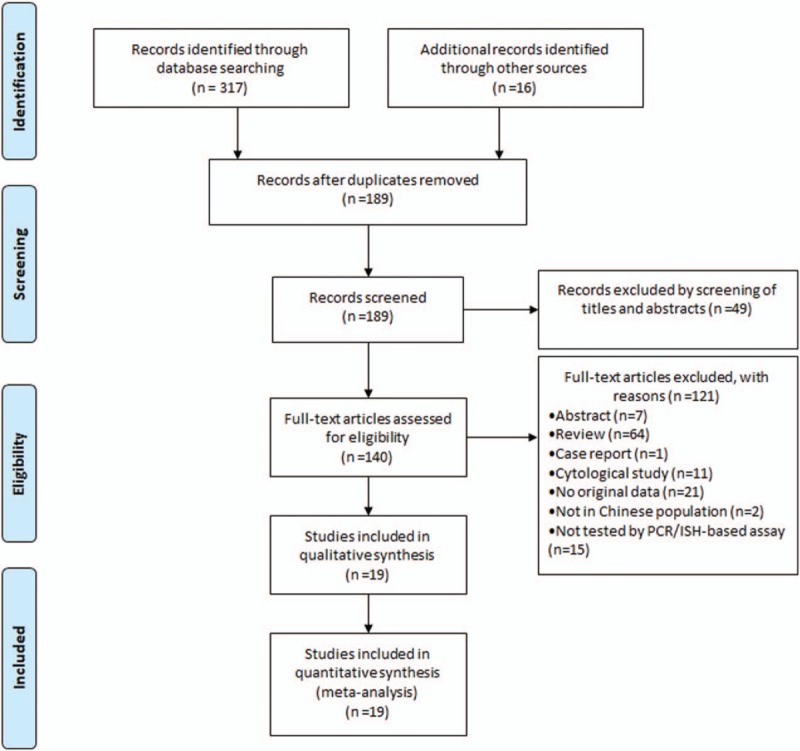
Flow diagram of systematic literature search on HPV-18 infection in head and neck cancer. HPV-18 = human papillomavirus 18.

Of 333 publications identified through an initial search of databases, 314 were excluded for reasons explained in Figure 1. So, 19 studies (7 in English and 12 in Chinese) were eligible and included in this meta-analysis.^[13–31]^ Individual characteristics of the included 19 studies were summarised in Table 1. The size of the study samples ranged from 7 to 333 cases of head and neck cancer (median = 75). Summing up the studies, a total of 1881 cases of head and neck cancer were identified. As shown in Table 1, the majority of studies were conducted in Eastern China (n = 12, 63.16%), with the remaining studies spanning 2 other regions of China as follows: 3 (15.79%) studies in Central China, 4 (21.05%) studies in Western China. 8 (42.11%) studies conducted in laryngeal cancer cases, 6 (31.58%) conducted in oral cancer cases, 3 (15.79%) conducted in oropharyngeal cancer cases, and the other 2 (10.53%) not reported the cancer sites. For HPV detection methods, 18 (94.74%) studies used PCR, 1 (5.27%) studies used ISH. 15 (78.95%) studies used FFPE, and 4 (21.05%) studies used FF. Besides, 8 (42.11%) studies used the gene detected from HPV E6/E7 region, and 4 (21.05%) used the gene detected from HPV L1 region.

**Table 1:**
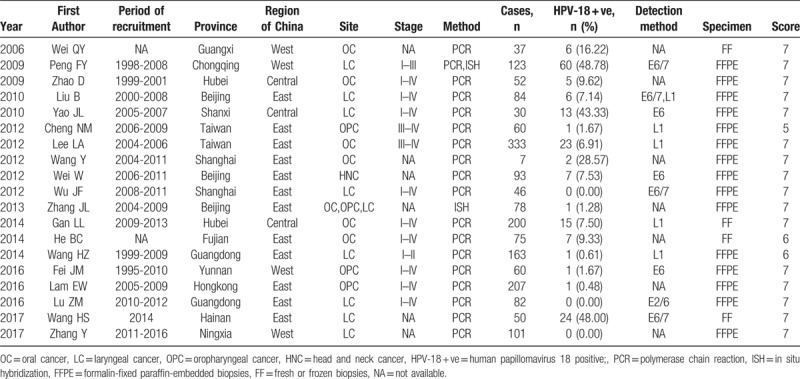
Studies included in the meta-analysis and their characteristics.

### 3.2 Meta-analysis of HPV-18 prevalence in head and neck cancer cases

**Figure 2:**
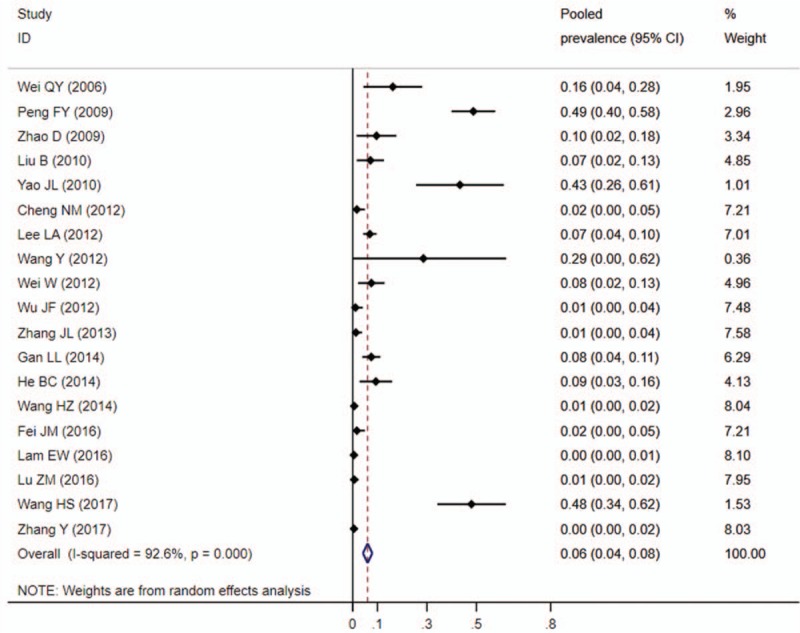
Forest plots of meta-analysis on HPV-18 prevalence in head and neck cancer tissue. HPV-18 = human papillomavirus 18.

Figure 2 was the forest plot illustrated the individual and pooled prevalence estimates derived from a random effect model analysis. In this study, the prevalence of HPV-18 ranged from 0.0% to 48.78%. The pooled prevalence for HPV-18 was 6.0% (95% CI, 4.1%–7.9%) in head and neck cancer cases in the Chinese population. Overall, high heterogeneity was observed in the studies included (*Q*-test *P*_heterogeneity_ <.001, I^2^ = 92.6%).

### 3.3 Subgroup analysis

Table 2 presents detailed results of subgroup analyses. As shown in Table 2, the highest pooled HPV-18 prevalence was observed in Central China (16.8%; 95% CI, 3.6%–30.0%), followed by that in Western China (15.3%; 95% CI, 5.2%–25.4%), and in Eastern China (3.3%; 95% CI, 1.6%–5.0%). Laryngeal cancer had the highest infection rate of HPV-18 (31.2%; 95% CI, 13.0%–49.4%), followed by oral cancer (7.2%; 95% CI, 3.9%–10.5%) and oropharyngeal cancer (0.6%; 95% CI, 0.0%–1.3%). Stratified analysis by published year showed that head and neck cancer before the year 2011 had the highest HPV-18 prevalence (24.4%; 95% CI, 7.0%–41.8%) as compared with the period from 2011 to 2015 (3.8%; 95% CI, 1.7%–5.8%) and the period from 2016 to 2017 (1.8%; 95% CI, 0.0%–3.9%). Stratified analysis by number of patients showed that head and neck cancer in less than 100 samples (6.6%; 95% CI, 3.8%–9.4%) had almost the same prevalence of HPV-18 compared with more than 100 samples (6.3%; 95% CI, 3.3%–9.4%). HPV-18 prevalence with PCR method was 12.6% (95% CI: 9.3%–16.0%). Hierarchical analysis by specimen types showed that head and neck cancer in FF tissue (18.7%; 95% CI, 6.2%–31.2%) had higher prevalence of HPV-18 compared with the FFPE tissue (4.3%; 95% CI, 2.5%–6.1%). Regarding the HPV-18 test methods, the prevalence of head and neck cancer in the E6/E7 region of HPV gene (29.5%; 95% CI, 15.6%–43.3%) was significantly higher than that in the L1 region (3.9%; %95 CI, 0.5%–7.4%). Stratified analysis by quality score showed that head and neck cancer in low-quality studies (2.2%; 95% CI, 0.0%–5.0%) had lower prevalence of HPV-18 compared with high-quality studies (7.5%; 95% CI, 5.1%–9.9%). In short, the estimated heterogeneity for studies included decreased to some degree but did not obliterate.

**Table 2:**
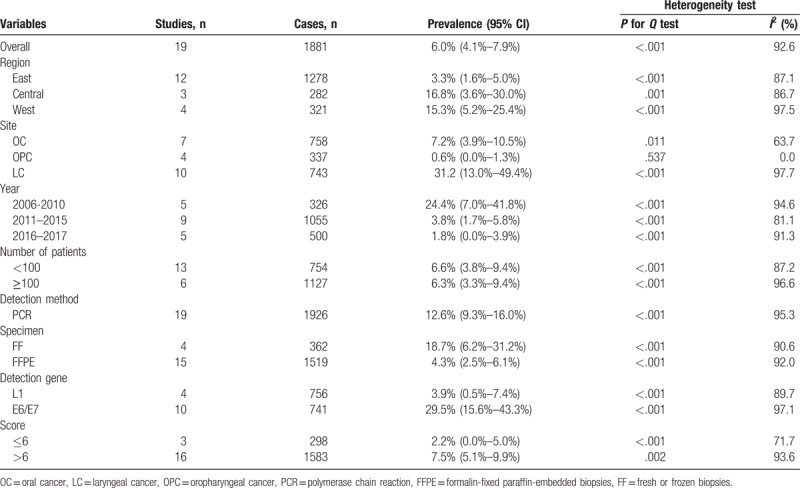
Results of subgroup analyses for HPV-18 prevalence in head and neck cancer lesion.

### 3.4 Publication bias

**Figure 3:**
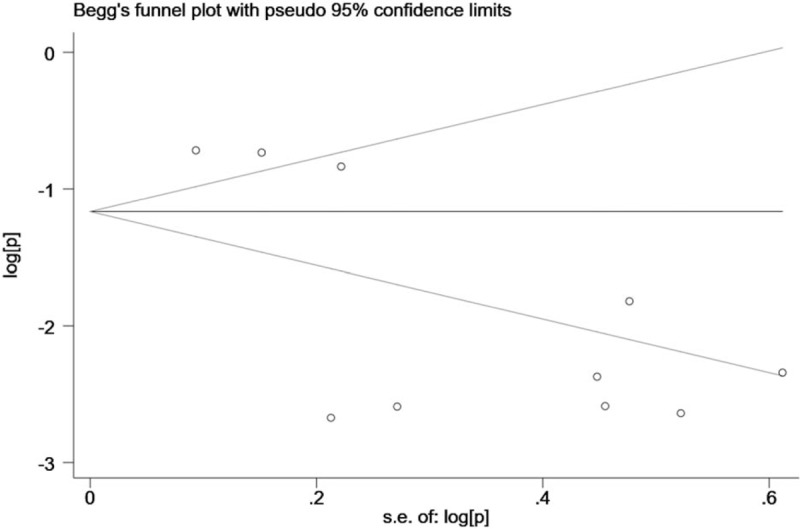
Funnel plots for publication bias.

There was no evidence of publication bias as demonstrated by the non-significant *P* values of Begg test (0.421), and the near-symmetric funnel plot (Fig. 3).

## 4 Discussion

China has the highest burden of head and neck cancer around the world, which was rational for studying the prevalence of certain types of HPV (type-18) in China for the highly lethal cancer. As we know, this is the first meta-analysis to exploring the prevalence of HPV-18 in Chinese head and neck cancer tissues. The results of the meta-analysis showed that more than 6% cases of head and neck cancer harbored HPV-18, indicating a high level of HPV 18 infection in head and neck cancer cases of China. Our results were little higher than the global prevalence, especially for laryngeal cancer.^[32]^

Characterizing the HPV-18 prevalence in head and neck cancer is an important preliminary step in assessing the relationship between HPV-18 and head and neck cancer. Estimates of the HPV prevalence in various cancer sites of head and neck cancer vary considerably. Our meta-analysis showed that laryngeal cancer had the highest HPV-18 prevalence (31.2%), followed by that in oral cancer (7.2%). Oropharyngeal cancer seemed not easily to be infected by the HPV-18, with the lowest prevalence (0.6%). Interestingly, the prevalence of HPV-18 in oropharyngeal cancer and oral cancer were much lower than Asian average but much higher in laryngeal cancer.^[32]^ More cases involved and areas covered could be helpful in estimating the prevalence of HPV-18 in different head and neck cancer sites in the future.

The detection rate of HPV-18 DNA in FF tissue was found much higher than that in FFPE tissue, given significant DNA degradation could be observed in FFPE tissue.^[33]^ The HPV infection status was determined on FFPE tissue in the majority of included studies. It is known that the low detection rate of HPV DNA occur with the fabric of FFPE, especially when a long DNA fragment was amplified.

Some possible reasons for variation in HPV prevalence among studies include small study sizes, different HPV testing, inter-laboratory variability, and manipulation of specimens leading to contamination.^[34–36]^ In our meta-analysis, PCR and ISH were just used as HPV detection methods, which focused on the HPV DNA detection method. Besides, for better PCR and ISH sensitivity and specificity, the literature time was limited from January 1, 2006 to May 31, 2018. HPV, a double-stranded circular DNA virus, encodes early proteins (E1, E2, E5, E6, and E7) and late proteins (L1, L2, and E4) with about 8000 bp genome size.^[37,38]^ When stratifying the L1 and E6/E7 gene fragments, we found that, in E6/E7 gene fragment (29.5%), the detection rate of HPV-18 DNA was much higher than in L1 gene fragment (0.5%). This is mainly due to the disruption of L1 region when HPV is integrated into the host genome,^[39]^ which may be an important event in promoting and triggering head and neck cancer.

The highlights of this meta-analysis include a large sample size, both English and Chinese published studies are included with a strict inclusion criteria. By including English and Chinese studies, the selection bias caused by the publication language was avoided. Finally, by astricting studied published after 2006 and limiting PCR and ISH detection methods, we tried to minimize the HPV prevalence variation as much as possible.

However, the meta-analysis has several limitations. First, the studies included in the meta-analysis are heterogeneous, which could be explained by changes in the population, the cancer site, the year of publication, the number of patients, the HPV detection method, the sample collection method, and the sensitivity of HPV primer PCR different protocols. To solve this issue, the random-effects model was used in the meta-analysis to combine data if significant heterogeneity was found. We directly tested heterogeneity by describing the HPV-18 prevalence in head and neck cancer cases by study area, cancer site, year of publication, number of patients, method of HPV detection, types of specimen, and quality score. Of course, we have not been fully able to explain the heterogeneity. Even in stratified outcomes (for example, studies in different parts of China), the prevalence estimates are still uneven. Second, the study estimates may be biased because the accuracy of these estimates depends on the test method used and the type of HPV evaluated. That is, some studies use multiple probes or wide primers to detect multiple types of HPV, while other studies only detect HPV-18 type. Finally, the possibility of confounder cannot be ruled out. Limited studies have implicated the effect of age and smoking on the prevalence of HPV. We could not determine whether the variation of HPV prevalence was due to differences in environmental factors: age, sexual habits, smoking, alcohol consumption, and other ethnic and cultural differences, as little or no information on these potential confounders in the studies involved. Therefore, studies with good designs to explore HPV infection by major confounding factors are likely to be required in future studies.

In short, the current meta-analysis provides a quantitative estimate of HPV-18 prevalence in head and neck cancer lesions in China. Although this review is a preliminary step in assessing the relationship between HPV-18 and head and neck cancer in China, it may be useful to evaluate the effect of HPV-16/18 prophylactic vaccines against carcinogenesis in the future. Considering that HPV infection plays an important role in the tumorigenesis of head and neck, other scientific studies deserve to be done in the future.

## Author contributions

All authors have made substantial contributions to the conception and design of the study. LWG and FNY led protocol design, search, data extraction, statistical analysis, and manuscript drafting. YLY and PL contributed to search, data extraction, and manuscript modifications. XJZ, DFC, YL, and JW contributed to quality assessment and revision of the manuscript. LWG contributed to data interpretation and revision of the manuscript. All authors have reviewed and approved the final version.

**Conceptualization:** Yulin Yin, Lanwei Guo.

**Data curation:** Peng Li, Xiaojun Zhang, Yang Liu.

**Formal analysis:** Defeng Chen.

**Investigation:** Peng Li.

**Methodology:** Yulin Yin, Jian Wang.

**Software:** Jian Wang.

**Writing – original draft:** Funa Yang, Xiaojun Zhang.

**Writing – review & editing:** Lanwei Guo.
